# Calcified wall portal venous aneurysm: a case report

**DOI:** 10.11604/pamj.2016.25.93.9896

**Published:** 2016-10-18

**Authors:** Safouane Khairallah, Abdelmajid Elmansouri, Hicham Jalal, Mariem Ouali Idrissi, Najat Cherif Idrissi Ganouni

**Affiliations:** 1 Department of Radiology, Mohammed 6^th^ Teaching Hospital, Cadi Ayyad University, Marrakesh, Morocc

**Keywords:** Portal aneurysm, portal calcifications, ultrasond, Computed tomography

## Abstract

Portal vein aneurysms are extremely rare, less than 200 cases have been reported until late 2015. They are defined as a portal vein diameter exceeding 19 mm for cirrhotic patients and 15 mm in normal livers. Most patients are asymptomatic, but complications may occur. We report a case of a 68-year-old female admitted for etiological assessment of a portal hypertension revealed by an upper gastro intestinal bleeding, who was incidentally diagnosed with a portal vein aneurysm.

## Introduction

Venous aneurysms are uncommon, and have been reported to occur in most major veins. Portal localisation accounts for around 3% of all venous aneurysms [1]. It is defined as a vein diameter exceeding 19 mm in cirrhotic patients and 15 mm in patients with normal livers. The first case was described in 1956 and since then, less than 200 cases have been reported [2].

## Patient and observation

**Figure 1:**
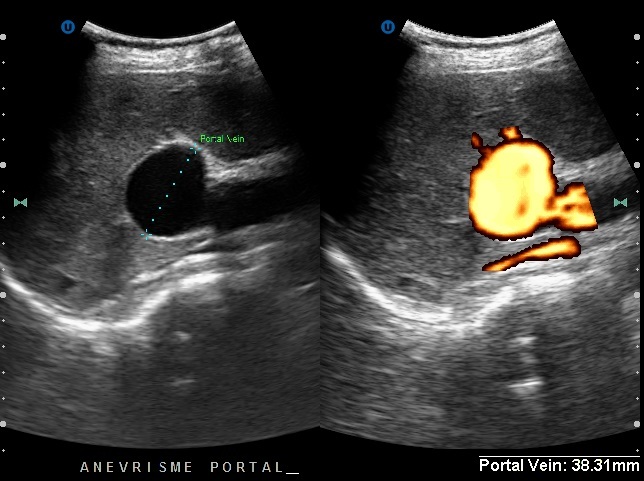
Portal vein presenting a fusiform dilatation in ultrasonography

**Figure 2:**
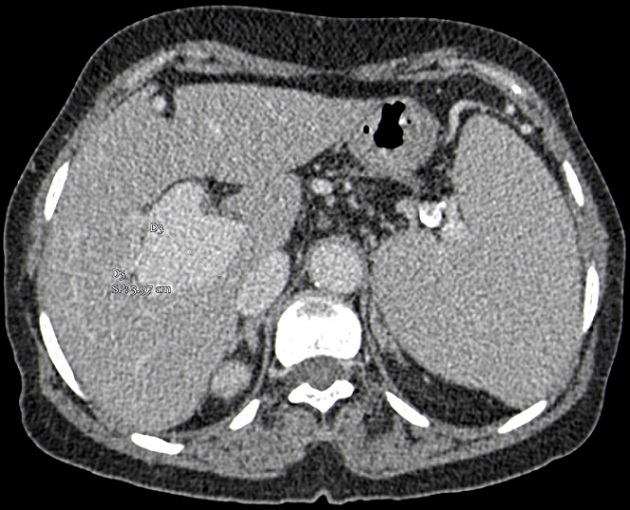
Axial reconstruction of a portal-phase angio-CT showing portal aneurysm located in the portal bifurcation

**Figure 3:**
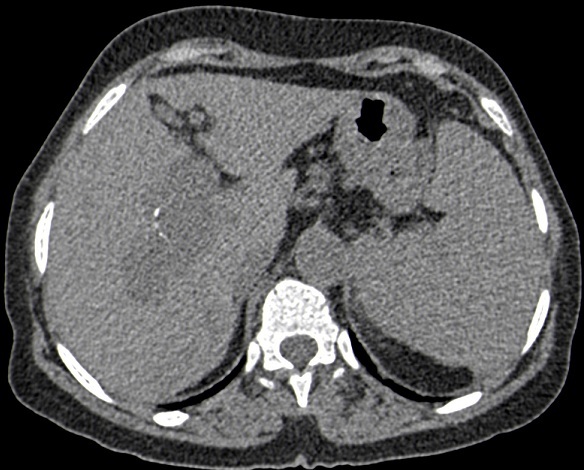
Thin wall calcification of the portal vein aneurysm

**Figure 4:**
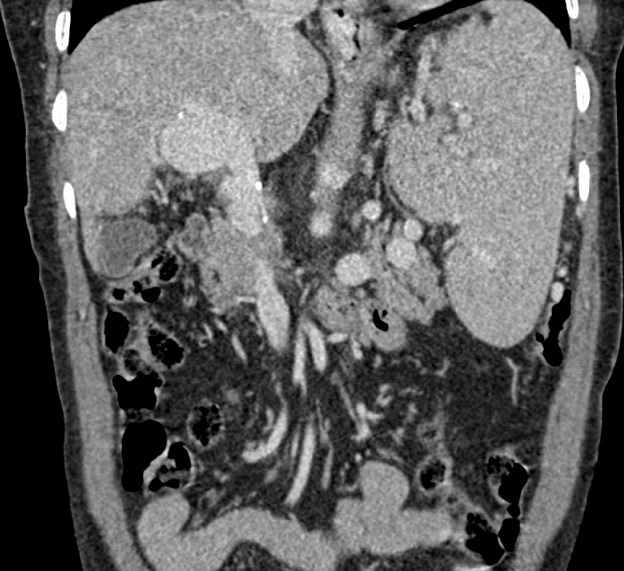
Coronal reconstruction of a portal-phase angio-CT showing portal aneurysm, thin wall calcifications appear in the portal trunk and the aneurysm

We report the case of a 68-year-old female with no past medical history, presented with an upper gastrointestinal bleeding and pain in left upper abdomen. Physical examination found a splenomegaly and signs of portosystemic collateral formation including abdominal wall dilated veins and rectal haemorrhoids. No biological abnormalities were noted besides those due to hypersplenism (anaemia and thrombocytopenia). The patient underwent upper gastrointestinal endoscopy that showed stage III oesophageal varices with portal hypertensive gastropathy and red signs. Ultrasonography revealed a fusiform dilatation of the portal bifurcation measuring 40 mm in diameter with dilated branches; no signs of portal thrombosis were detected (Figure 1). The portal Doppler study showed a normal hepatopetal flow. Other signs of portal hypertension were found, including splenomegaly, repermeation of the umbilical vein and a splenorenal collateral circulation. A complementary angio-CT allowed a better assessment of the portal system that contained some thin calcifications in the aneurysmal wall and the main portal trunk (Figure 2, Figure 3, Figure 4). Other signs of portal hypertension were noted such as oesophageal and gastric varices, parietal collateral circulation associated to ultrasonography findings. Liver cirrhosis was confirmed by transcient elastometry and biopsy.

## Discussion

Aneurysms in the visceral veins are uncommon, but they have been described in most major veins [1]. Portal aneurysms represent less than 3% of all visceral aneurysms, Barzilai and Kleckner first described it in 1956 [2]. PVA is defined as a portal vein diameter exceeding 19 mm in cirrhotic patients and 15 mm in normal livers, its etiologies are not clearly established, but it is considered either congenital or acquired [3]. Ninety-six reports of PVA were published between 1956, the year it was first reported, and late 2015. These reports included 190 patients, with a mean age of 56, ranging from 0 to 89 years old [4]. PVA may be an incidental finding since one third of the patients were asymptomatic and 50% of them presented non-specific abdominal pain. Symptoms associated with portal hypertension are also reported.The two most frequent locations were the main portal trunk and the portal bifurcation, as it is the case of our patient; other locations include spleno-mesenteric confluence, right and left portal branches [5]. None of the cases reported mentioned aneurysmal wall calcifications. The majority of patients were explored at the time of diagnosis with abdominal Doppler ultrasound and computed tomography. Ultrasound is suitable to evaluate the patency and the blood flow in the aneurysm [6]. Portal venous-phase CT with 3D reconstructions allows a better assessment of the location. Portal hypertension is often associated with portal aneurysms, it is described as a cause and a consequence of portal vein aneurysms, although its etiology remains unclear. With portal hypertension, there is intimal thickening and compensatory medial hypertrophy of the portal vein. With time, the medial hypertrophy is replaced by fibrous tissue that weakens the tensile strength of the vein wall, making it susceptible to aneurysmal dilatation [6]. Calcifications in the portal vein or its tributaries are rare. They usually occur in patients with long-standing portal hypertension too, regardless of underlying etiology. Calcifications within the intima and media of the vein may result from mechanical stress [7].

## Conclusion

Portal vein aneurysm and portal calcifications are both rare entities that are associated to portal hypertension, they may both be incidentally found in an ultrasonography or an angio-CT scan of a patient suffering from a portal hypertension, regardless of underlying etiology.
